# Supplementary material for: A Global Screen for Assembly State Changes of the Mitotic Proteome by SEC-SWATH-MS
Source: Cell Syst. 2020 Feb 26;10(2):133–155.e6. doi: 10.1016/j.cels.2020.01.001 (PMC7042714; doi:10.1016/j.cels.2020.01.001)

O96017 | CHK2_HUMAN | CHEK2 CDS1 CHK2 RAD53
Monomer MW [kDa]: 60.915 Monomer expected elution fraction: 45

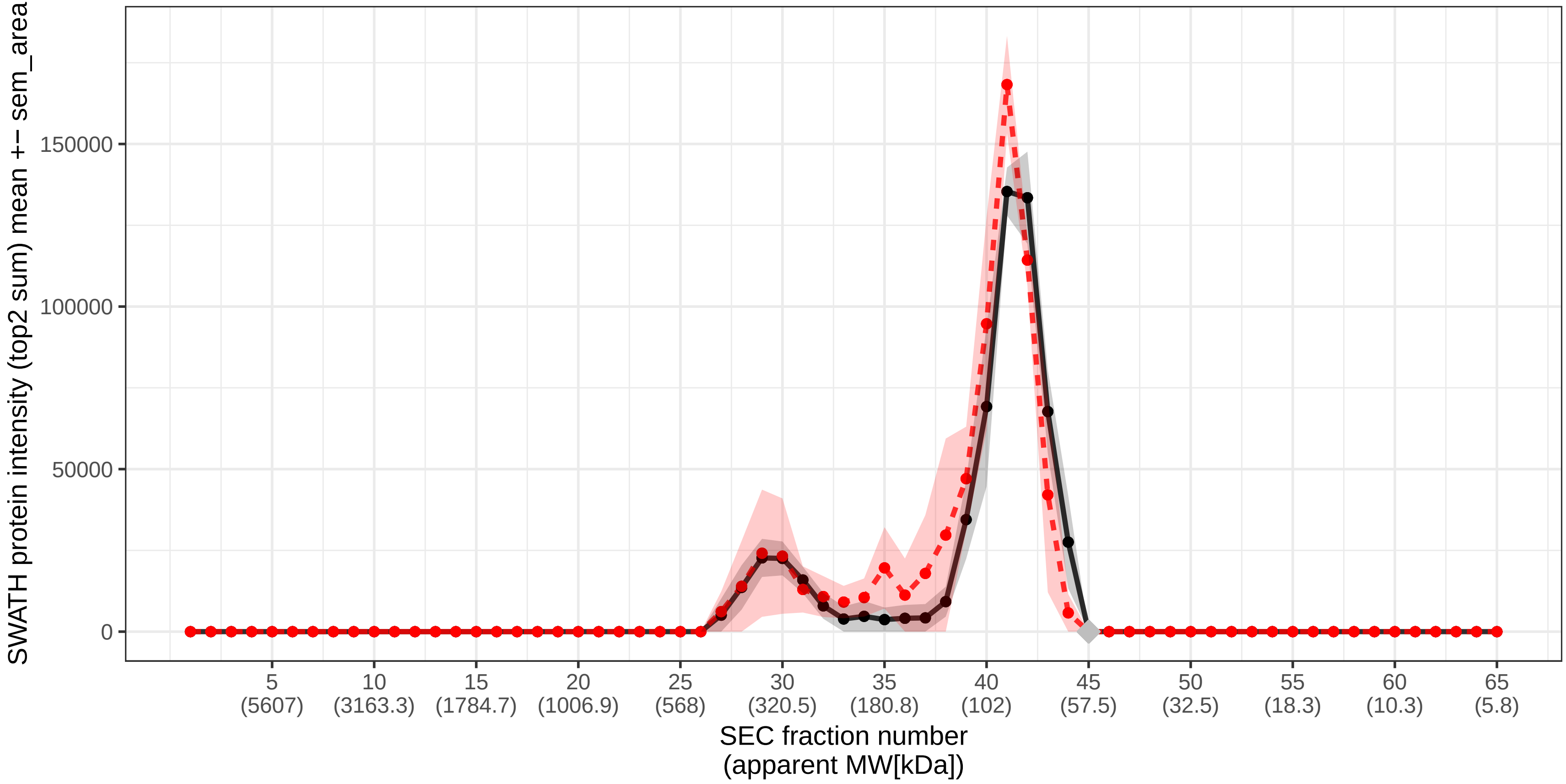

Supplement: Data S1. SEC-SWATH-MS Protein Chromatograms, Related to Figure 1 [file mmc6.zip › SECchrom_O96017_CHK2_HUMAN_CHEK2_CDS1_CHK2_RAD53.pdf]
